# Healthcare provider experiences of deploying a continuous remote patient monitoring pilot program during the COVID-19 pandemic: a structured qualitative analysis

**DOI:** 10.3389/fdgth.2023.1157643

**Published:** 2023-06-28

**Authors:** Juliana Pugmire, Matt Wilkes, Adam Wolfberg, Nicole Zahradka

**Affiliations:** ^1^ Clinical Research, Current Health Ltd., Edinburgh, United Kingdom; ^2^ Clinical Research, Current Health Inc., Boston, MA, United States

**Keywords:** digital health, remote patient monitoring, telehealth, telemedicine, qualitative research, implementation science, pilot programs, feasibility studies

## Abstract

**Objective:**

To describe the healthcare provider (HCP) experience of launching a COVID-19 remote patient monitoring (CRPM) program during the global COVID-19 pandemic.

**Methods:**

We conducted qualitative, semi-structured interviews with eight HCPs involved in deploying the CRPM pilot program in the Military Health System (MHS) from June to December 2020. Interviews were audio recorded, transcribed, and analyzed thematically using an inductive approach. We then deductively mapped themes from interviews to the updated Consolidated Framework for Implementation Research (CFIR).

**Results:**

We identified the following main themes mapped to CFIR domains listed in parentheses: external and internal environments (outer and inner settings), processes around implementation (implementation process domain), the right people (individuals domain), and program characteristics (innovation domain). Participants believed that buy-in from leadership and HCPs was critical for successful program implementation. HCP participants showed qualities of clinical champions and believed in the CRPM program.

**Conclusion:**

The MHS deployed a successful remote patient monitoring pilot program during the global COVID-19 pandemic. HCPs found the CRPM program and the technology enabling the program to be acceptable, feasible, and usable. HCP participants exhibited characteristics of clinical champions. Leadership engagement was the most often-cited key factor for successful program implementation.

## 1. Introduction

### 1.1. Background

As the SARS-CoV-2 (COVID-19) pandemic took hold, healthcare organizations, along with many other sectors, faced an unprecedented challenge (1). Many countries, cities, and hospitals were not equipped to deal with the number of people requiring assessment and care and were uncertain as to how the demands might escalate as the pandemic evolved (2). Routine and elective services were canceled or delayed, while healthcare providers (HCPs) focused their attention on patients with COVID-19 (3). Lawmakers, insurance companies, and medical organizations took action to quickly eliminate many of the regulatory and financial barriers to utilization of digital health technologies (DHTs) (4). These technologies, which enabled health systems to provide virtual care, were adopted in settings with the infrastructure, resources, and will to push them forward (5, 6). From March 2020, there was a 20-fold increase in the use of telemedicine (5). COVID-19 pushed these technologies to the forefront, accelerating their adoption at scale (7). Unsurprisingly, not all of these initiatives were successful. Therefore, as virtual care continues to evolve, it is essential that successful programs are evaluated and their lessons should be parsed and disseminated (4, 5).

The COVID-19 remote patient monitoring (CRPM) pilot program implemented within the Military Health System (MHS) in the United States was an example of a highly successful virtual care program (8).

### 1.2. Military Health System and the COVID-19 remote patient monitoring pilot program

The MHS is one the most complex and largest healthcare systems in the United States that is tasked to deliver healthcare services to almost 10 million uniformed service members, military retirees, and families (9). The Defense Health Agency (DHA) is a combat support agency that manages clinical and business operations across the MHS (10).

Before the COVID-19 pandemic, the MHS had some capabilities for telehealth (11). These capabilities might have been manually driven, for example, a telephone call to check a patient's blood pressure with a device issued by the MHS. Leadership within the DHA Virtual Medical Center submitted a proposal to the DHA to request funds through the Coronavirus Aid, Relief, and Economic Security (CARES) Act (12) to support a pilot program focused on remote patient monitoring. The purpose of the pilot program was to effectively and safely manage COVID-19 outside of treatment facilities for patients who did not require in-home skilled nursing care (13). In this way, they hoped to reduce the need for staffing, relatively keep healthier patients safely in their homes, and open bedspace for high-risk patients who needed to be in the hospital (13).

The pilot proposal was approved, and the Virtual Medical Center launched a CRPM program in May 2020. Military treatment facilities (MTFs, i.e., military hospitals or clinics) were given information about the pilot program and invited to participate if they could show HCP buy-in by providing one lead physician and one lead nurse to run the project at their facility. The pilot phase of CRPM began in August 2020 with 10 participating MTFs from all three military services (i.e., air force, army, navy) across the United States, enrolling patients hospitalized by COVID-19 infection. From March 2021, additional populations, including post-surgical and acute medical patients, were also enrolled in the CRPM program. The data collection period of the CRPM pilot program ran from 7 June 2020 to 7 December 2020.

### 1.3. Current Health

**Figure 1:**
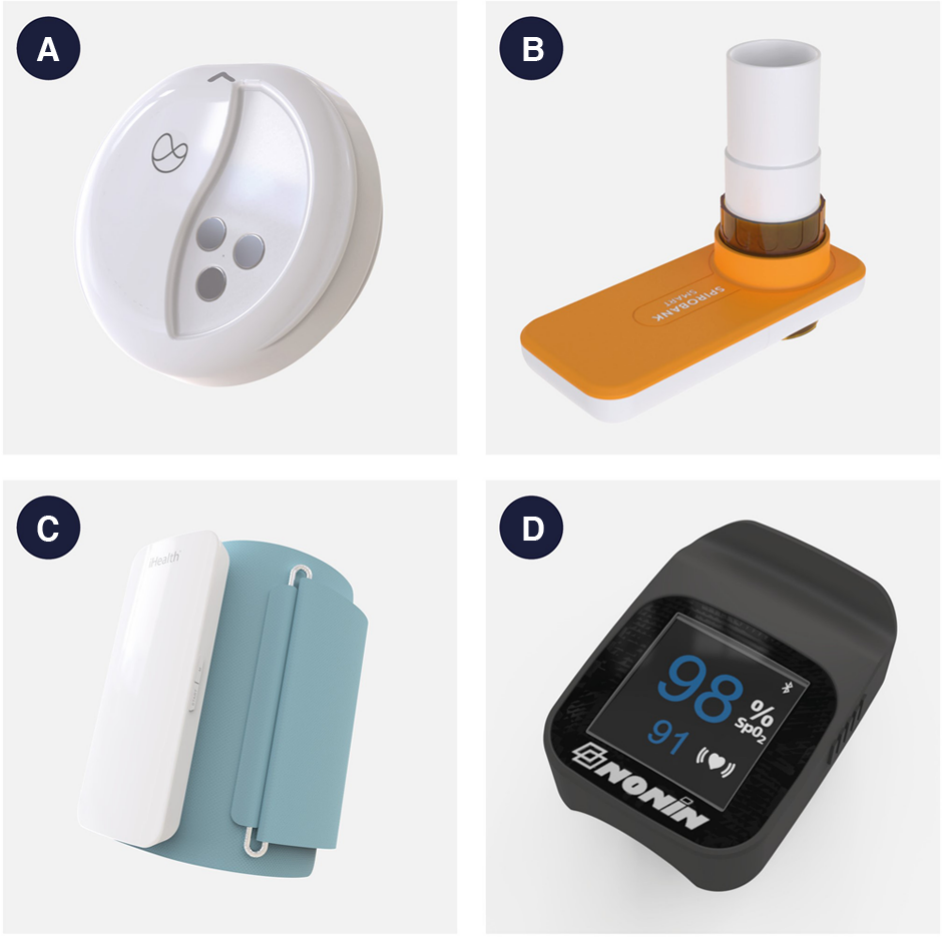
Pictures of the Current Health wearable device and peripherals. (**A**) Current Health wearable device. The device is fitted in an armband and worn on the upper arm. Approximately 4.6 cm diameter and 1.7 cm depth. (**B**) Spirometer. Approximately 10.9-cm-length, 9-cm-tall mouthpiece. (**C**) Blood pressure cuff. Device is approximately 10 cm wide × 15.5 cm long × 1.9 cm deep and attaches to fabric cuff. (**D**) Pulse oximeter. Approximately 4.6 cm wide × 6.9 cm long × 3.3 cm deep.

**Figure 2:**
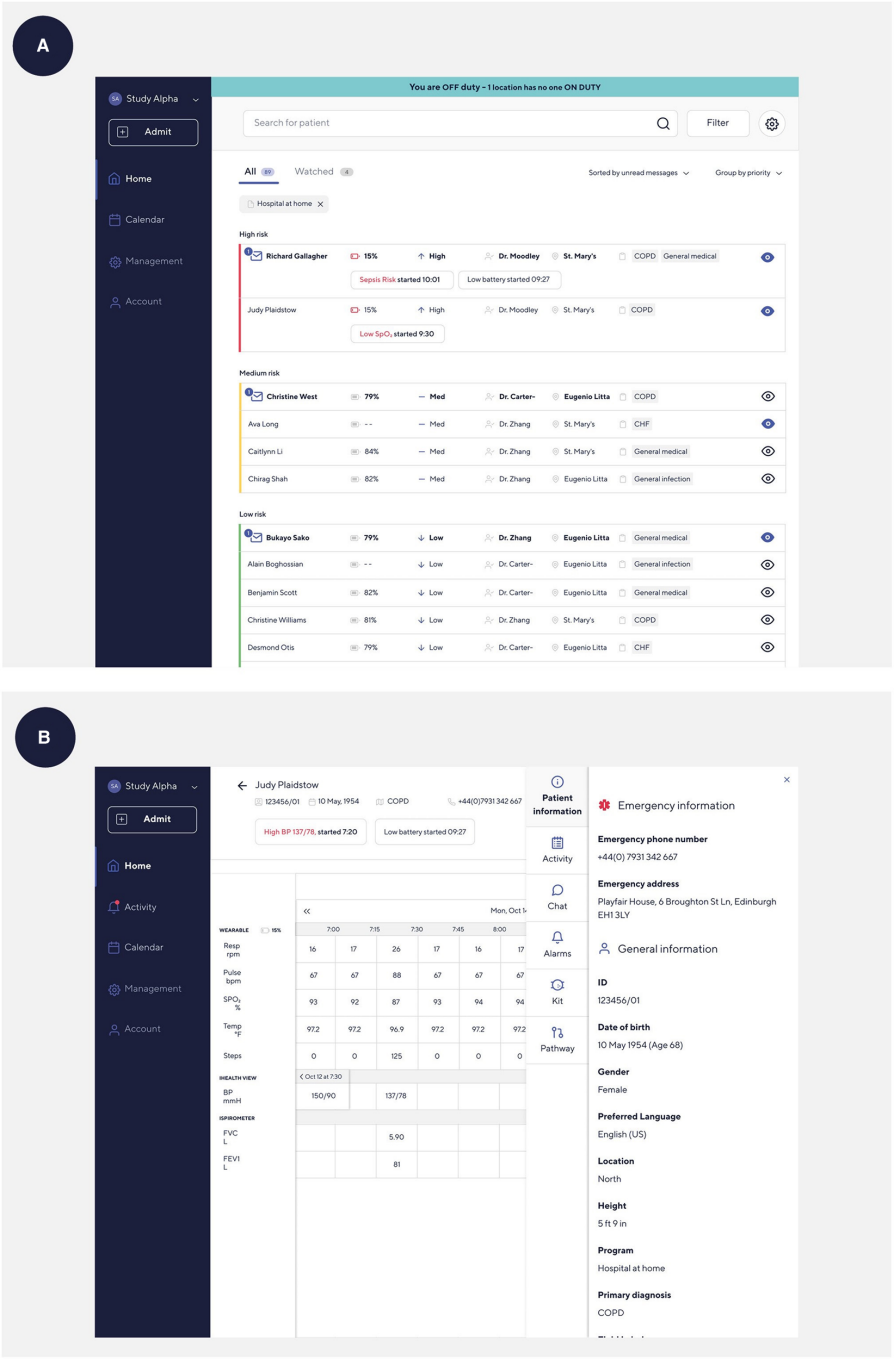
Screenshots of the Current Health clinical dashboard. (**A**) Home screen on the Current Health clinical dashboard. (**B**) Patient screen on the Current Health clinical dashboard.

The MHS chose the Current Health platform for their virtual care program (Current Health Inc., Boston, MA USA) after undergoing a rigorous market analysis and vetting process. The Current Health platform enables healthcare organizations to deliver healthcare services at home for different clinical conditions (e.g., COVID-19, heart failure, obstetric care, oncology) and acuity levels. It includes an FDA-cleared small, round, wearable device worn on the upper arm and integrated peripherals to monitor vital signs (e.g., oxygen saturation, pulse rate, respiration rate) (14). It incorporates a tablet to connect with HCPs via video calling and to deliver surveys or reminders to a patient to take measurements. It also includes a hub for internet connection, so patients without home Wi-Fi can still be included in RPM programs, thus making care delivery more equitable. HCPs have access to a Current Health clinical dashboard, such as the well-known hospital observation chart, to review patient data and are alerted if a patient's vital signs go out of normal parameters. This enables HCPs to check on patients via the tablet, ask a patient to come into a clinic or hospital, or escalate care through emergency services if necessary. It means that some patients can be safely discharged from hospital care while continuing to be monitored from the comfort of home. Figures 1 and 2 provide photos and dimensions of the wearable device; some peripherals such as a blood pressure cuff, spirometer, and pulse oximeter; and a screenshot of the HCP clinical dashboard.

We conducted a qualitative study, interviewing personnel involved with developing and implementing the CRPM pilot program to understand the participant experience of deploying the program and what might have made it a success.

### 1.4. Digital health technologies in clinical care

DHTs offer substantial opportunities to improve clinical care, research, and public health. They can give HCPs a more holistic and longitudinal view of a patient's health trajectory and patients more control and understanding of their own health (15). From diagnostic tools (16, 17), clinical study endpoints (18), informed consent (19), study recruitment (20, 21), and public health outcomes (22), DHTs are making a difference in the lives of patients, HCPs, research scientists, and research participants. It is in this climate that the World Health Organization recently released its first guidelines on digital health interventions (23).

Digital health has a broad scope and according to the FDA includes “categories such as mobile health (mHealth), health information technology (IT), wearable devices, telehealth and telemedicine, and personalized medicine” (24). DHTs utilize software, sensors, and platforms aimed at the general population (“health and wellness” devices) through clinical care (devices that are FDA-approved or cleared on a par with hospital technology) (24).

While HCPs and patients have given high scores for the perceived usefulness and ease of use of wearable sensors that provide continuous monitoring (25), DHTs bring challenges of privacy, regulation, and data quality (26, 27). They can take up time if the HCP has to manually enter more electronic health record data (28). They can be intrusive (29, 30) or inadvertently widen health disparities by not accommodating vulnerable populations such as older adults (31, 32); this can be mitigated by conscious steps to widen access to technology (33). Despite complex challenges around adoption of DHTs, they are rapidly being integrated into healthcare delivery (26).

### 1.5. Adopting and implementing healthcare programs: what it takes

Implementing change, whether involving DHTs or not, can take considerable resources (34). Many and perhaps most efforts to implement change fail and are more likely to fail without considering the factors critical to the success of implementation (35, 36). Factors identified in the literature were both multilevel and interactive. They included the individual, team, organization, larger system levels or context, and innovation (36–39). Leadership, culture, team development, and information technology are also important to consider when exploring the program implementation (36). For example, the implementation climate is a reflection of an organization's willingness to reward and cultivate innovation and has an impact on program success (40).

Key factors associated with successful program implementation are explored, described, expanded on, and defined in the updated Consolidated Framework for Implementation Research (CFIR) (41) which provides “a menu of constructs that have been associated with effective implementation” (42). The CFIR is based in large part on the work of Greenhalgh et al.'s “How to Spread Good Ideas: A systematic review of the literature on diffusion, dissemination and sustainability of innovations in health service delivery and organisation” (43, 44). CFIR is an implementation determinant framework, meaning that it is designed to identify and describe barriers and facilitators to implementation outcomes (44, 45). In the implementation science literature, clinical champions have been shown to be important to the successful implementation of practice changes (46). These were individuals in an organization that motivated others, had a responsibility to encourage change, and used their position and knowledge to influence change (e.g., virtual healthcare program) (37). Within CFIR, clinical champions are built into the individuals domain under various leadership constructs (e.g., leaders, implementation leads, innovation deliverers) with a characteristics subdomain (need, capability, opportunity, motivation) (41). We used the CFIR constructs to frame and understand the barriers and facilitators identified by the HCPs responsible for creating and implementing the CRPM pilot program.

### 1.6. Research objectives

The overall objective of this qualitative study was to explore the experience of HCPs in launching the CRPM program. Our research objectives were to:

1. Understand the processes involved in operationalizing and using the CRPM pilot program.
2. Explore feasibility, acceptability, and usability of the CRPM pilot program among HCPs.
3. Explore barriers and facilitators of deploying an RPM program during the global COVID-19 pandemic.

## 2. Methods

### 2.1. Population, recruitment, and procedure

Our population of interest included HCPs (inclusive of their administrators) who helped stand-up and deploy the CRPM pilot program rolled out to hospitals within the MHS between June and December 2020. Interviews took place between November 2021 and March 2022. We used information power (47) to guide our sample size, and no one was excluded from participation within this population.

Participants were initially recruited from collaborators within MHS and thereafter from interviewees themselves using a convenience and snowball sampling strategy (48). Twenty-four participants were offered the opportunity to participate in a one-to-one interview via email and were told the reasons they were invited to participate in this research. One site was not granted clearance to participate in the interviews (Naval Medical Center in Portsmouth). Eight participants agreed to participate.

Participants that agreed to an interview were remotely consented. They could sign the informed consent form ahead of speaking with the researcher or go over it at the start of the call. They could opt out of audio recording the interview, but none chose this option.

### 2.2. Interview schedule and interviewing

Based on previous experience developing interviews for understanding engagement with digital technology and exposure to the CFIR (44) and the Theoretical Domains Framework (TDF) (49), the study team designed the interview schedule to understand processes, feasibility, acceptability, usability, highlighting barriers, and facilitators around the successful program implementation of the CRPM pilot program during the COVID-19 pandemic (e.g., CH training, impact on job performance, patient reception to technology use, overall attitude toward digital health, etc.). The topic guide/interview schedule was designed before any interviews were conducted and shared with MHS personnel for approval. One-to-one, in-depth interviews were conducted by JP by phone at a pre-arranged, mutually convenient time and lasted up to an hour. Table 1 provides an overview of the interview schedule.

**Table 1:** Interview schedule overview.

Interview schedule overview
Intro/HCP background experience and context in which HCP has been using CH.
HCP perception and user experience of the CH platform and getting set up.
How the CH platform may have affected the HCP ability to do his/her job, especially during COVID-19.
HCP perception of the patient experience using CH during COVID.
HCP attitude toward RPM and technology adoption.

JP had an email communication with two participants who invited her and the Current Health research team to conduct this qualitative study. These communications were for ensuring that correct protocol was followed through study setup. There was no relationship established between the remaining six participants prior to study commencement. All participants knew that JP was a research scientist employed by Current Health. At the time of the study, JP was a Senior Clinical Research Scientist at Current Health. She has over 10 years of experience conducting interviews for mixed-methods and qualitative research, holds a DrPH and MPH in epidemiology, and is a female.

### 2.3. Data collection

Participant interviews were recorded using a laptop application (Windows Voice Recorder, Microsoft Corporation) and a handheld digital recorder as a backup. Interviews were anonymized and transcribed using a third-party transcription service. This transcription was then checked by the researcher that conducted the interviews (JP) using Trint software (Trint Ltd., London, UK) when necessary. If there were any words or phrases that were unintelligible to the researcher (JP), a second researcher (JLT) checked the audio. In this way, the transcribed data were rigorously assessed for accuracy. Familiarization with the data began at this early stage. JLT was an employee of Current Health at the time the study was conducted. No field notes were made, no repeat interviews were conducted, and no transcripts were returned to participants for review or comment. However, participants were available to answer questions about their interview after it took place.

### 2.4. Data analysis

JP coded the data using QSR International NVivo Qualitative Data Analysis Software version 12 (50). The data were inductively analyzed following the steps outlined by Braun and Clarke for reflexive thematic analysis, specifically (1) familiarization of data (JP conducted the interviews, listened to the interviews, and checked the transcriptions of the interviews for accuracy), (2) generating initial codes (data were inductively coded to identify different meanings; codes were shifted as more interviews were analyzed to differentiate range of meanings), (3) searching for themes (JP looked for shared meaning among code labels and used visual mapping for theme development; candidate themes were generated after the initial analysis of four interviews), (4) reviewing themes (JP used candidate themes to code the final interview transcripts and visual mapping for theme review), (5) defining and naming themes (themes were refined, ensuring that they were meaningful for the data as a whole, and then deductively mapped onto CFIR constructs to provide an interpretative lens to examine findings), and (6) producing the report (used quotes to provide evidence for themes linked to CFIR constructs) (51, 52).

## 3. Results

We followed the Consolidated Criteria for Reporting Qualitative Research (COREQ) checklist for reporting results (53) (Appendix A).

Because we had a small sample and individuals could easily be identified, we aggregated participant characteristics and attributed quotes to the HCP role level to protect anonymity. There were two nurses, three nurse leads, two physicians, and one HCP administrator interviewed (four males, four females). “Nurse lead” was defined as a leadership role, either within the CRPM program or in their department.

We identified and explored in-depth the following themes: internal and external environments, processes around implementation, the right people, and program characteristics. We mapped these themes to the updated CFIR domains and constructs, and these results are presented in Table 2.

**Table 2:** Themes.

Theme	Description of theme	CFIR domains and constructs
External and internal environments	Factors involved in spurring the creation of a new healthcare delivery service relating to environment, political will, legislation, regulation, and technological conditions	Outer setting domain
		Local condition
		Policies and law
		Inner setting domain
		Tension for change
	Organizational characteristics associated with the successful implementation of the CRPM pilot program	
Processes around implementation	Identifying and understanding the factors involved in strategically implementing or adopting new programs or procedures: planning, engaging, deploying, reflecting and evaluating	Implementation process domain
		Planning
		Engaging
		Reflecting and evaluating
The right people	Qualities and characteristics of the individuals that were part of implementing a successful CRPM program, including HCPs, industry partners, and types of patients enrolled into the CRPM pilot program	Individuals domain
		Innovation deliverers
		Implementation leaders
		Innovation recipients
Program characteristics	The qualities and characteristics of the intervention and the technology that made it a success, including an improved sense of connection between nurses and their patients. It also includes participant thoughts about the innovativeness of the CRPM pilot program and how RPM, hospital at home, and platforms such as CH fit into the future of healthcare	Innovation domain
		Innovation adaptability
		Innovation relative advantage
		Innovation cost
		Innovation design

### 3.1. External and internal environments: outer setting and inner setting domains

This theme relates to the updated CFIR constructs of local conditions and policies and laws, set within the outer setting domain (41). These two constructs deal with the environmental (COVID-19 pandemic), political/legislative/regulatory (creation of the CARES Act), and technological (lack of technology to address patient needs) conditions that enabled the delivery of the CRPM pilot program. The theme also relates to the inner setting domain construct of tension for change as highlighting that the current situation was intolerable (not being able to care for patients) and needed to change. The COVID-19 pandemic was identified by all participants as an essential factor in the development of the CRPM pilot program within MHS. The CARES Act (12) provided a quick funding stream (local conditions, policies and law), COVID-19 surges made it apparent that current healthcare capabilities could not meet the demand (local conditions, tension for change), and political will was united in solving problems created by the pandemic (local conditions). It was evident in these interviews that the status quo was simply not up to the task of handling the increased volume of patients within the MHS and that the pandemic forced action and implementation that otherwise might have taken much longer (tension for change).

*At the time [preparing for COVID], we didn't have anything. The remote patient monitoring capabilities we had were very rudimentary.*

Nurse Lead

*I think remote monitored programs had been tried before, but there just hadn’t been any incentive to do it or the need.*

Physician

*The CARES Act allowed us to be able…to do a very rapid funding request and get that approved, literally in a matter of weeks, which is unheard of, to ask for multimillion-dollar contracts and be able to actually keep those within the period of several weeks*.

Physician

COVID-19 was such a significant disruptor; it set in motion the political will (local conditions) to drive requirements for care (policies and laws). Once the requirements were in place, the processes and solutions developed around them.

*A requirement makes people have to do it, and then actually it can help drive the resources to support it*.

Physician

There were organizational characteristics identified by participants that affected the implementation of the CRPM pilot program. The lead MTF was one of the largest military medical centers in the Department of Defense, had army leadership, and was a tri-service facility, meaning that they had army, navy, and air force personnel. Participants said that within the MHS, risk was generally not well-tolerated and communication could be a major issue because of the size of the organization.

### 3.2. Processes around implementation: implementation process domain

The CFIR describes the implementation process domain as the “activities and strategies used to implement the innovation” (41, 42) which the participants discussed at length when describing how they set up the CRPM pilot program. The constructs that related to the themes we found in this work were (1) planning (the steps and processes associated with planning to implement the innovation such as identifying responsibilities, setting milestones, and defining measures of success and goals), (2) engaging (encouraging participation in implementation or delivery of the innovation), and (3) reflecting and evaluating (discussing and examining the information related to the success of the implementation).

#### 3.2.1. Planning

The planning and creation stages involved identifying problems and solutions in the earliest stages of program development and deployment. One participant shared an anecdote about the COVID-19 surges that were headed their way based on what the world had seen in Italy and New York. He had a contact with the Chief of Infectious Disease at a major academic medical center in New York who agreed to talk with him about lessons learned, and it was this conversation that got him thinking about an RPM platform as a possible solution. Another participant recounted a colleague asking whether there were any technological solutions to help with the approach of a COVID-19 surge. Participants also knew that they needed to plan for adequate staffing.

*I think it was probably around April of 2020 right after COVID-19 started spreading across Europe, the Chief of Pulmonary/Critical Care Medicine…came to me and said, ‘You know, we’ve seen what's happening in Europe and Italy right now, ICUs are becoming overwhelmed with patients. Do we have any type of technology or capability where we can keep patients out of the hospital to thereby prevent the spread of infection internally to the organization and to protect these patients who don’t have it from getting it if they’re immunocompromised?’ Basically, how can we keep patients in their homes, decrease the need for staffing and open up bedspace for those who really need to be in the hospital?*

Nurse Lead

*Just having talked to a couple of the military treatment facilities that were interested in participating, we knew that if we asked them to provide organic nursing support to monitor their patients that it was doomed to fail*.

Nurse Lead

#### 3.2.2. Engaging

Leadership sought buy-in from HCPs within MTFs that could act as clinical champions for the CRPM pilot program as well as from the individuals responsible for integrating the CRPM pilot program into the hospital workflow. Training was also used to ensure competence and engagement in the innovation deliverers. All training was virtually conducted because the pandemic made in-person training untenable. However, there was equipment onsite so staff could see it and handle it in-person, and this was an important part of feeling comfortable and engaged with the technology.

*Our actual training was fairly fast. When we had meetings, we were in person, and we were able to touch and feel and kind of play with the mechanisms of the Current Health kit.*

Nurse

#### 3.2.3. Reflecting and evaluating

Besides training, HCPs acknowledged that it took some time to recognize how to efficiently run the CRPM pilot program. Nurses and physicians had to reassess their roles and interactions in the virtual environment. Learnings, problems, and solutions were discussed in regular meetings with CRPM project leads, and issues that arose were shared with leadership.

*The nurses seemed to be trained well to kind of know when to start reaching out to us [physicians],* vs. *me just kind of…looking at the data, and saying, ‘okay, what am I seeing?’. But that just became inefficient, and so I started relying more and more on the nurses, to start highlighting important stuff*.

Physician

In the first month, when only a handful of patients had been enrolled in the CRPM pilot program, the teams involved had an opportunity to see the Current Health technology and implementation teams in action and began gaining confidence that the system was working.

*We had two or three patients that the nurses were monitoring at home and felt that they were decompensating and got them back to a facility to make sure that they were getting care and what they needed. So, I think that validated that the technology works and that our nurses understood what their role was, to make sure that if the patient was decompensating, we had a good mechanism in place to get them the care that was necessary to prevent further decompensation*.

Nurse Lead

### 3.3. The right people: individuals domain

The CFIR identifies many types of roles within the individuals domain that contribute to successful program implementation. For example, the innovation deliverers deploy the innovation to recipients known as innovation recipients. There are also constructs for leaders with varying levels of authority, decision-making capabilities, and informal influence. Implementation leads are individuals who lead the innovation implementation and can also be thought of as clinical champions. The CFIR goes onto categorize characteristics of roles into (1) need (the innovation will address deficits related to the individual's survival or well-being), (2) capability (the knowledge and competence to fulfill the role), (3) opportunity (the power and scope to fulfill the role), and (4) motivation (the individual's commitment to fulfilling the role) (41).

Participants talked about HCP and leadership qualities that contributed to successful implementation of the CRPM pilot program. These qualities included enthusiasm for and belief in the program (need, motivation) and an ability to anticipate problems, manage expectations, and persist through them (capability). Finding the right people came up repeatedly in interviews as one of the most important factors associated with implementation. Participants spoke about finding (a) the right HCPs or innovation deliverers to deliver new programs using new technology and the right industry partners to collaborate with, (b) engagement and support from leaders and champions, and (c) the right patients or innovation recipients to be enrolled in the CRPM program. Qualities and characteristics of these “right people” are explored in more detail below and linked to CFIR constructs explained above.

#### 3.3.1. The right HCPs and industry partners: innovation deliverers

Enthusiasm for the CRPM pilot program and innovation was evident in all participant interviews (motivation). They brought into the pilot's proof of concept and believed in the technology as a solution for both current and future patients and healthcare settings (need). They believed that CH helped them “complete the mission” by providing the right “constellation of capability” in caring for patients.

*I think what has been unique about Current Health is that it's integrated not just real-time by physiologic monitoring—or near real time—but also the ability to pair that with devices which add collateral information…And then at the most acute level, be able to facilitate video communication in real time with the patient. So, that kind of constellation of capability*.

Physician

It was clear that HCP participants deeply cared for their patients and had patient needs in mind while designing this program (motivation). They felt that the Current Health platform allowed them to improve their primary objective of improving the patient experience. The technology allowed them to help a broader range of patients, providing them with a safety net and allowing the patient to feel empowered by being more involved in their own care. This sense of purpose was coupled with an ability to persevere through the growing pains of implementation (capability).

*I'm able to really help a broader range of people… And overall, our goal is always to help more people. We're able to get them the help that they need and get them back into the hospital faster than they probably ever would have in an emergency, be their safety blanket and help their anxiety. I mean, that peace of mind goes a long way*. *So we had to go back…there were times that we had to go back to the drawing board and say, ‘okay…you can’t see this, let's try doing it this way.’*

Nurse

### 3.4. Nurse lead

#### 3.4.1. Leaders: leaders and implementation leads

Participants talked about leadership engagement and the implementation climate at MHS. They all agreed that leadership buy-in was critically important. Furthermore, the leadership included HCPs in discussions around the CRPM program development and implementation from the earliest stages. This in turn facilitated acceptance from the HCPs creating a collaborative environment that supported the emergence of clinical champions (implementation leads).

*What was great about this was our leadership actually asked the nurses what we thought about, what we could do better or how we should do it…So I think we all played a crucial role in giving our two cents as far as the set-up process and the process itself of getting this thing going, because I don't think we had anything really to go off of. We were the first to ever get this done*.

Nurse

A key part of successful program implementation was finding clinical champions to lead, build, and communicate. Clinical champions are a known critical component of successful program implementation and can be mapped to implementation leads in the updated CFIR. These implementation leads were baked into the process of implementing the CRPM pilot program.

*There was an agreement that if an MTF [military treatment facility] was going to participate they had to provide one lead provider and one lead nurse to run the project at their facility, and that was critical because we knew again that if we didn't have physician and nursing buy-in to support this at the functional and leadership level, that it was not going to be successful*.

Nurse Lead

This participant went on to explain that while they were contracting for the technology (Current Health) and the nursing staff, they simultaneously formed a clinical working group (capability, motivation). He said that one person took the lead on this because of his extensive connections in pulmonary departments across facilities (opportunity). Other participants identified leads that made a tremendous contribution to the CRPM pilot program implementation and saw them as critical to the program's success.

*He was amazing. He is the biggest cheerleader for Current Health. He thought of everything, and he really pushed it out and really just got providers excited and let them know the potential that remote patient monitoring could bring to better patient outcomes*.

Nurse

For an intervention as complex as the CRPM pilot program, there were a huge number of variables that affected success. When something went wrong, was it the technology, the HCPs using the technology, or the patients using the technology? This puzzle of possibilities necessitated a strong partnership with the company providing the CH platform and technology. Participants talked about the qualities and characteristics of their CH colleagues that factored into program implementation which track with the CFIR characteristics subdomain constructs. Current Health innovation deliverers were seen as being quick to respond to requests (opportunity, capability), being open and receptive to feedback (motivation), wanting to make improvements (motivation), and always putting the patient first (need, capability) that were identified as important qualities in CH colleagues.

*Any time we had a ticket put in for any tech issues, regardless of whether or not our patients had COVID, I mean [CH] staff was pretty much on it. They responded quickly…and we appreciate that. The consistency is very much appreciated, especially in an unknown time, and the COVID itself is stressful on the patient coming home and being at the hospital is stressful enough*.

Nurse

*I think the best part of it was [CH had] a really good team that wants to make sure that our patients are safe and that we can always keep an eye on the patients*.

Nurse

### 3.5. The right patients: innovation recipients

At the beginning of the deployment, the type of patient enrolled into CRPM could not require high levels of care and in fact needed to be well enough that they were close to discharge and did not require nurse visits at home. However, they also had to be willing to use the CH kit. While many patients were thrilled to be able to recuperate at home and have the security of an HCP watching over their vital signs, there were some limiting factors. One of the limiting factors around participation was the patient's willingness to engage with technology, and this could vary based on age, clinical condition, or support from more technologically savvy family members. The hurdle of the initial setup could be challenging at a time when a patient was feeling ill and vulnerable. In other words, a patient's capability and motivation were important factors around program adoption (capability, opportunity, motivation).

*A lot of the older folks would look at that jumble of wires and would immediately just become scared…And you'd tell them, you've got an iPhone in your hand. If you have an iPhone in your hand, and you know how to plug something into a wall, you can do this, I promise you can do it. Some people would refuse to do it after they'd looked at the equipment and say this is going to be too complicated for me*.

Nurse Lead

### 3.6. Program characteristics: innovation domain

The innovation domain in the updated CFIR focuses on factors related to the intervention or thing being implemented, in other words, the innovation. Innovation adaptability reflects the extent to which the innovation was able to be modified or tailored to fit the local context. Innovation relative advantage reflects whether the innovation is better than other alternatives or current practice. Innovation cost and innovation design are about the innovation's affordability and how well it is packaged, assembled, and presented. In line with these CFIR constructs, the CRPM pilot program was adaptable and could be integrated with current workflow (innovation adaptability), had relative advantage over the status quo (innovation relative advantage), showed return on investment (innovation cost), and could be learned quickly and easily (innovation design).

The CRPM pilot program implemented by the MHS was viewed by participants as an innovative and cutting-edge approach to handling the increase in patient volume caused by COVID-19. Many spoke about the problems that already existed around access to healthcare and saw that RPM and hospital at-home programs, which have seen enormous growth during the COVID-19 pandemic, become part of the healthcare landscape.

*We're really pushing the envelope, not just within our own community, but this was the first time that we were doing this within a military facility, but we were doing it really kind of at the leading edge of what was even being done in civilian medicine*.

Physician

The CRPM pilot program showed that it had innovation adaptability, as it was repurposed for new sites, new use cases, and new staffing capabilities. It was successfully incorporated into a facility's existing workflow. Adaptability is one of the reasons the CRPM pilot program was able to be used across different MTFs and branches of the military and later for different use cases like COPD or heart failure.

*…it [CH platform] incorporates with the current platforms that we have… new technology can be incorporated into the current workflow*.

HCP Administrator

*No one in the DOD [Department of Defense] was set up to use this [Current Health platform] and I think that multiple people in military treatment facilities came up with their own way of, ‘this is how we’re going to deliver the devices, these are the types of population of patients we’re going to use it on.’*

Nurse Lead

The adaptability of alarm thresholds proved critical to the program's success. HCPs were alerted if a patient's vital signs went out of pre-set parameters, setting in motion a chain of events ranging from calling the patient to asking the patient to go into a hospital or clinic or initiating emergency responses. Those alarming thresholds were initially set more conservatively than was recommended by medical personnel from Current Health. One participant talked about the consequences of this decision, why they took the course of action they did, and the subsequent decision to return to recommended thresholds.

*I think we've all become very used to and very good at looking at the alarm system…I do think we were very conservative, meaning that we were very generous and had wide alarm rates… But we felt at least at first, that was the right way to go and to over-activate and we certainly did over-activate. And we've seen that that went down pretty quickly from our nurses [laughs], that they were being…basically burdened with alarm fatigue as a result of the distinction*.

Nurse

One of the strongest reasons for implementing the CRPM pilot program was that it offered a substantial and essential relative advantage over keeping patients in the hospital (unfeasible during the pandemic) or only having intermittent vital signs data if patients were sent home (dangerous if a patient quickly decompensated). It did this by providing continuous monitoring *outside* the hospital which was better than current practice (innovation relative advantage). There is a real advantage to seeing trends in patient data vs. spot checks that makes RPM a part of healthcare that gives a more holistic view of a patient's health.

*I think the biggest thing is, I've learned a snapshot of vital signs is not necessarily a good indicator of how the patient is truly doing or even just what the patient is reporting is not always the best indicator of what is actually happening*.

Nurse

*…we needed to be able to provide a safety net for patients that were being discharged from the medical, surgical environment during…COVID. We needed a way to monitor those patients to make sure that they were okay, and that they didn't have a recurrence of symptoms or worsening of symptoms. And we needed to be able to move them out quicker. And this [CH platform] allowed us to do that*.

Physician

One unanticipated finding of this study found that nurses reported an improved sense of connection with their patients (innovation relative advantage), not *despite* providing remote care but *because* of providing remote care.

*Honestly, I would tell them this is by far the most engagement that I feel like I've had with patients…and so for a lot of us, we went into nursing because of education, and this gives us that opportunity to do that, and then some*.

Nurse

A major factor in any intervention will be its cost and return on investment (innovation cost). The MHS was collecting data to understand return on investment and cost savings of the CRPM pilot program and CH platform.

*…we were still able to show a significant return on investment within the first month of it going live. I mean, we were decreasing in-patient bed days, we were keeping patients out of the ER, we were reducing readmissions, we were decreasing average length of stay, you know?*

Nurse Lead

Another key characteristic that was vital to success was the ability for HCP users to quickly learn the technology and continue to use it with ease. Learning new DHT can be time-consuming for HCPs, but these participants reported that the Current Health platform was easy to learn and use (innovation design). This required the CH platform and kit to be well designed and easy to assemble and present.

*It was pretty intuitive for the information that was being provided, it was just a matter of figuring out how it was organized, and what the interface could do*.

Physician

## 4. Discussion

We explored HCP experiences in launching a COVID-19 remote patient monitoring pilot program during a global pandemic. Guided by our research objectives and the CFIR framework, we asked about processes, feasibility, acceptability, usability, barriers, and facilitators in implementing virtual healthcare with new technology during a stressful time. While understanding how HCPs dealt with these changes, we also wanted to understand what made this program successful (8).

### 4.1. Understanding the processes

The creators of the CRPM pilot program took a multilevel approach and considered the individual-, departmental-, institutional-, and national-level factors at play which is supported in the literature as being essential for program success (36). While planning, they sought input and buy-in from HCP users, leadership at the MTF and executive levels, and sourced national funding streams made available from the CARES Act.

Not only did the CRPM program developers seek buy-in from their stakeholders and HCP users but made sure that they did this at the earliest stages of development (26). Sharma et al. state that “close and early collaborations between stakeholders will be required to ensure that digital health technologies not only improve outcomes but add value to healthcare systems, decrease cost, and improve quality of care” (26). Beyond identifying stakeholders at an early stage, it was also pointed out that program implementers needed to ensure that stakeholders will be available during deployment.

As the program was implemented, all eight participants talked about the various ways that outcomes, success, and patient satisfaction were monitored. They spent a good amount of time planning the implementation and engaging stakeholders and were in a regular state of reflecting and evaluating implementation and program success, all CFIR constructs shown to be associated with high success implementations (54). They were continually looking for unanticipated barriers and had a process in place to deal with them. When problems arose, they would feed these back to the clinical working groups to identify solutions. Monitoring progress this way is known to be an important part of the implementation process (44).

Another key aspect of successful implementation processes is the close and early collaboration between the technology developers and clinicians (26). Participants in this study were all on a first-name basis with CH colleagues and spoke highly of their willingness to receive feedback and make requested improvements. There was a close working partnership throughout the pilot program.

### 4.2. Feasibility, acceptability, and usability

The CRPM pilot program and CH platform were adaptable, relatively advantageous to the status quo, and able to integrate with current workflow and hospital technology, were quickly learned and easily used, and showed return on investment as early as the first month of deployment. Relative advantage is a construct shown to be associated with a high level of success implementations (54). Overall, participants reported good innovation design including high ease of use and usefulness for the CH platform, kit, and technology, and this is an important component of DHT adoption and the success of interventions utilizing DHTs (55, 56). There were technical aspects of using the CH platform that were challenging and required troubleshooting. But participants believed that this was part of the process of standing up a new program and using new technology. They believed that the program and technology were so feasible, acceptable, and usable, and they wanted to expand it to different use cases, such as chronic obstructive pulmonary disease or heart failure.

Participants also believed that the CH kit was feasible, acceptable, and usable for most of their patients, the innovation recipients. Patients were overall pleased to be able to recover from home while being monitored and having the security of HCP eyes on their health status. However, there were some patients that were daunted by the technology, and there were times HCPs had to troubleshoot and identify family members or friends of the patient that could offer support for CH kit utilization once the patient was discharged.

The most surprising aspect of this research was that HCPs reported an enhanced sense of connection because of the CH platform and not simply despite it. From the nurses' points of view, this made them more interested in CRPM program involvement. There is some evidence that mHealth apps can have a positive effect on the patient–provider relationship (57, 58), and Areia et al. found that monitoring of the vital signs could be a way for HCPs to engage and connect with the patients (59). However, this research is relatively new and complicated when considering where and how it can be applied in practice (60).

### 4.3. Barriers and facilitators

Interestingly, while the COVID-19 pandemic made many things so much harder, it was frequently cited as the main catalyst for creating and sustaining the CRPM pilot program. Many participants believed that had COVID-19 not happened, they would not have had the funding, opportunity, or leadership drive to make it happen. COVID-19 meant that traditional methods for patient care were not up to the task of caring for the surge in patient volume. Finding solutions became a worldwide necessity as evidenced by the removal of regulatory barriers around virtual care and telehealth (61).

The MHS is large, mature, and composed of semi-autonomous departments and, as a result, would be predicted to assimilate innovations more readily (43, 62). Because of the COVID-19 pandemic, there was tension for change within the MHS, resulting in a rapid procurement of funding and personnel to help address the gaps in care that were fast approaching.

It was clear that study participants felt valued within this organization and supported through the work of creating and implementing the CRPM pilot program. Feedback loops between leadership, physicians, and nurses were reported by everyone, and HCP users most “on the ground” had feelings of ownership and pride for the work they were doing. Every participant strongly believed that leadership buy-in and top–down support was one of the key factors that was critical to successful program implementation. Indeed, leadership engagement, cross-boundary working relationships, and innovation-related communications have been shown to be associated with high implementation innovations (54).

While we did not set out to explore anything specifically around the concept of clinical champions, it was clear from the beginning of the interview process that we were in fact dealing with clinicians that fit the definition of implementation leaders or “clinical champions.” Identifying whether a program has a clear clinical champion or not is a key activity of assessing the implementation process (44). Participants all acknowledged the importance of finding the right people to make the CRPM pilot program happen, and indeed, clinical champions have been described in the literature as being essential for successful process implementation (63). Clinical champions often select themselves by volunteering for programs, and we saw that in this study. Champions and implementation leaders were asked to volunteer and were selected well before the CRPM intervention was deployed. It is especially important that clinical champions are allowed to self-select or are carefully selected (64). There is further evidence that front-line nurses in particular are an essential influential factor in the implementation of practice change (65) and nurses within this study were part of the development and decision-making from the beginning.

Beyond acknowledging that champions were important for implementation, participants spoke about the qualities of these “right people,” e.g., champions as well as HCPs. Broadly speaking, the characteristics and behaviors of clinical champions can be condensed into three main factors: expressing enthusiasm and confidence about the success of the innovation or program, persisting under adversity, and getting the right people involved (66, 67). The participants themselves showed a great degree of enthusiasm for the CRPM pilot program and strongly believed in the intervention and technology as a solution to keeping patients safe during the pandemic. In other literature, a focus on the innovation recipients (i.e., patients) was associated with high success implementations (54).

Participants were clear about the program goals and managed expectations around the inevitable wrinkles that were part of deployment.

Barriers to adoption of DHTs by HCPs can mainly be categorized into technical factors around security, individual factors around technology literacy, and health systems factors such as economics and policies (68). In line with other literature (56, 68), the security of RPM was discussed by participants who believed that standards were being met or exceeded in the CRPM pilot. Alarm fatigue can also be a major barrier to DHT adoption by HCPs (59), but this was resolved by changing the alarming thresholds.

### 4.4. Generalizability

While this research involved a military population in the United States, the main recommendations and learnings (Table 3) are not specific to military facilities or military personnel and could theoretically be generalizable to any facility that was large, mature, and composed of semi-autonomous departments, as many healthcare facilities are. The literature cited throughout the paper, and especially the “Discussion” section, are not based on military populations. One possible difference may be that an MTF, composed of a military population used to responding to orders, may have better or faster reactions to top–down directives than civilian healthcare facilities. However, if a healthcare facility is large and mature, top–down directives are likely to exist, and the factors that can make top–down directives effective, like a sense of relative advantage among innovation deliverers, are likely still important for successful implementation (54). The qualities associated with clinical champions and leaders successfully implementing a program will not be particular to military operations but include non-military operations as well, as evidenced by the literature cited in this paper.

**Table 3:** Key recommendations and learnings with reference to CFIR constructs in parentheses.

• Remote patient monitoring can be done safely and can be beneficial for the right patient • Find the right people: ◦ The right personnel/staff (implementation leaders and innovation deliverers)—and enough of them—to implement the program, use the technology and equipment, and enroll patients. These “right personnel” can ideally see the relative advantage of the innovation. ◦ The right personnel have a range of characteristics and skills identified as essential to successful program deployment and implementation. All of them have skills and competence (capability), power (opportunity), and commitment to fulfill their role (motivation). They also have: ▪ Flexibility ▪ Critical thinking ▪ High tolerance for adapting and pivoting quickly ▪ Acceptance of inevitable problems and ability to problem-solve ◦ The right leaders that ask for team input during development and provide top–down support to help build a program ◦ The right patients for the program (innovation recipients) • Identify stakeholders early, and ensure that they will be available at the time of program deployment

## 5. Recommendations

Table 3 summarizes recommendations and learnings from the CRPM pilot program.

## 6. Limitations

Like many studies involving in-depth interviews, the sample was restricted to participants that were willing and able to grant an interview. A military setting can make it difficult to secure interviews as active-duty military have duty assignments away from home or move jobs more frequently than a civilian population. The nurses involved in the CRPM pilot program were not active duty, however, but contracted out and that also could make it difficult to connect with possible interviewees. This was likely one reason that we had eight participants out of the 24 invitations we sent to individuals. Four of those invitations were for an MTF that did not allow HCPs to participate in this research. This could mean that the participants willing to respond to our requests for interview had more positive feelings about the CRPM program than individuals that did not respond, and indeed, our participants were all directly involved with implementing the CRPM pilot program. However, we had access to two participants that helped develop this study and assisted with finding interviewees. Because of this, we know that even though we could not secure interviews with some individuals invited for interview, they nonetheless were champions of the CRPM program.

Our methodology could have been strengthened by designing follow-up with our participants to validate our interview schedule. However, recruiting HCPs or other busy professionals is very challenging (69, 70). We believed that our chances of successful recruitment would decrease if we asked participants to give more than 1 h of their time for an interview. We were supported in this line of thinking by our MHS colleagues that invited us to conduct this research.

The study had a good balance of nurses and physicians and one HCP administrator, and we believe that we had reasonable information power (47) though our findings may have been expanded with higher numbers. We believe that the research could also have been benefited by interviewing personnel from MTFs that declined to participate in the pilot program. However, the eight participants that agreed to an interview were likely some of the more enthusiastic developers of the program.

## 7. Future research

Finding a way to make the technology unintimidating to patients that may otherwise struggle with technology will further reduce health disparities associated with access to this type of care. One of our most interesting and unanticipated findings was that in this pilot program DHT enhanced a sense of connection with patients as reported by HCPs. We believe that future research could focus on what aspects of the CH platform contributed to this enhanced connection and whether patients experience this as well. Intuitively, the research team believed that technology could feel cold and that being remotely monitored might reduce a sense of connection as it surely reduced face-to-face contact in this intervention. There may be more to learn about the kind of care an HCP delivers via DHTs and what kind of “website manner” leads to better connection (71).

## 8. Conclusions

We sought to understand the HCP experience of launching a successful continuous RPM program during the global COVID-19 pandemic. The CRPM pilot program and CH platform were found to be acceptable, feasible, and usable. HCP participants showed the qualities of enthusiasm, persistence with problem-solving and expectation management, ability to involve the right people in the CRPM pilot, and a belief in the CRPM program. Leadership buy-in was the most often-cited key factor for successful program implementation.

## Data Availability

The data sets presented in this article are not readily available because while transcripts are anonymized, some participants may be identifiable because of the information and details discussed. Requests to access the data sets should be directed to juliana.pugmire@currenthealth.com.

## References

[1] Onyeaka H Anumudu CK Al-Sharify ZT Egele-Godswill E Mbaegbu P. COVID-19 pandemic: a review of the global lockdown and its far-reaching effects. Sci Prog. (2021) 104:368504211019854. 10.1177/00368504211019854 34061685 PMC10454957

[2] Organisation for Economic Co-operation and Development. The territorial impact of COVID-19: Managing the crisis across levels of government. OECD). Available at: https://www.oecd.org/coronavirus/policy-responses/the-territorial-impact-of-covid-19-managing-the-crisis-across-levels-of-government-d3e314e1/ (Accessed April 17, 2023).

[3] Elezkurtaj S Greuel S Ihlow J Michaelis EG Bischoff P Kunze CA Causes of death and comorbidities in hospitalized patients with COVID-19. Sci Rep. (2021) 11:4263. 10.1038/s41598-021-82862-5 33608563 PMC7895917

[4] Roberts ET Mehrotra A. Assessment of disparities in digital access among medicare beneficiaries and implications for telemedicine. JAMA Intern Med. (2020) 180:1386–9. 10.1001/jamainternmed.2020.2666 32744601 PMC7400206

[5] Cantor JH McBain RK Pera MF Bravata DM Whaley CM. Who is (and is not) receiving telemedicine care during the COVID-19 pandemic. Am J Prev Med. (2021) 61:434–8. 10.1016/j.amepre.2021.01.030 33781622 PMC7936544

[6] Hollander JE Carr BG. Virtually perfect? Telemedicine for COVID-19. N Engl J Med. (2020) 382:1679–81. 10.1056/NEJMp2003539 32160451

[7] Golinelli D Boetto E Carullo G Nuzzolese AG Landini MP Fantini MP. Adoption of digital technologies in health care during the COVID-19 pandemic: systematic review of early scientific literature. J Med Internet Res. (2020) 22:e22280. 10.2196/22280 33079693 PMC7652596

[8] Walter RJ Schwab SD Wilkes M Yourk D Zahradka N Pugmire J Financial and clinical impact of virtual care during the COVID-19 pandemic: difference-in-differences analysis. J Med Internet Res. (2023) 25:e44121. 10.2196/44121 36630301 PMC9879318

[9] About the Military Health System. Military Health System. Available at: https://www.health.mil/About-MHS (Accessed April 17, 2023).

[10] Defense Health Agency. Military Health System. Available at: https://www.health.mil/About-MHS/OASDHA/Defense-Health-Agency (Accessed September 2, 2022).

[11] Madsen C Poropatich R Koehlmoos TP. Telehealth in the Military Health System: impact, obstacles, and opportunities. Mil Med. (2023) 188:15–23. 10.1093/milmed/usac207 36882030

[12] US Department of the Treasury. About the CARES Act and the Consolidated Appropriations Act. U.S. Department of the Treasury. Available at: https://home.treasury.gov/policy-issues/coronavirus/about-the-cares-act (Accessed August 30, 2022).

[13] COVID-19 remote patient monitoring pilot marks initial successes. Available at: https://myarmybenefits.us.army.mil/News/COVID-19-remote-patient-monitoring-pilot-marks-initial-successes (Accessed April 18, 2023).

[14] Current Health. Remote Patient Monitoring. Available at: https://currenthealth.com/platform (Accessed January 6, 2022).

[15] Dunn J Runge R Snyder M. Wearables and the medical revolution. Epub ahead of print 27 September 2018. doi: 10.2217/pme-2018-0044 10.2217/pme-2018-0044 PMC12294383 30259801

[16] Isakadze N Martin SS. How useful is the smartwatch ECG? Trends Cardiovasc Med. (2020) 30:442–8. 10.1016/j.tcm.2019.10.010 31706789

[17] Turakhia M. Apple Heart Study: Assessment of Wristwatch-Based Photoplethysmography to Identify Cardiac Arrhythmias. Clinical Trial Registration NCT03335800, clinicaltrials.gov. Available at: https://clinicaltrials.gov/ct2/show/NCT03335800 (Accessed June 29, 2022) (2020).

[18] Snipelisky D Kelly J Levine JA Koepp GA Anstrom KJ McNulty SE Accelerometer-measured daily activity in heart failure with preserved ejection fraction. Circulation. (2017) 10:e003878. 10.1161/CIRCHEARTFAILURE.117.003878 28588021 PMC5634329

[19] Grady C Cummings SR Rowbotham MC McConnell MV Ashley EA Kang G. Informed consent. N Engl J Med. (2017) 376:856–67. 10.1056/NEJMra1603773 28249147

[20] McConnell MV Shcherbina A Pavlovic A Homburger JR Goldfeder RL Waggot D. Feasibility of obtaining measures of lifestyle from a smartphone app: the MyHeart counts cardiovascular health study. JAMA Cardiol. (2017) 2:67–76. 10.1001/jamacardio.2016.4395 27973671

[21] Loren DeVito P. MJFF's Targeted Social Strategy Accelerates Research Participation | Parkinson's Disease. Available at: https://www.michaeljfox.org/news/mjffs-targeted-social-strategy-accelerates-research-participation (Accessed July 1, 2022).

[22] Odone A Buttigieg S Ricciardi W Azzopardi-Muscat N Staines A. Public health digitalization in Europe. Eur J Public Health. (2019) 29:28–35. 10.1093/eurpub/ckz161 31738441 PMC6859512

[23] World Health Organization. Recommendations on digital interventions for health system strengthening (2020). Available at: https://www.who.int/publications-detail-redirect/9789241550505 (Accessed July 25, 2022). 31162915

[24] US Food and Drug Administration. What is Digital Health? FDA. Available at: https://www.fda.gov/medical-devices/digital-health-center-excellence/what-digital-health (Accessed July 7, 2022).

[25] Claudio D Velázquez MA Bravo-Llerena W Okudan GE Freivalds A. Perceived usefulness and ease of use of wearable sensor-based systems in emergency departments. IIE Trans Occup Ergon Human Factors. (2015) 3:177–87. 10.1080/21577323.2015.1040559

[26] Sharma A Harrington RA McClellan MB Turakhia MP Eapen ZJ Steinhubl S Using digital health technology to better generate evidence and deliver evidence-based care. J Am Coll Cardiol. (2018) 71:2680–90. 10.1016/j.jacc.2018.03.523 29880129

[27] Lancet T. Is digital medicine different? Lancet. (2018) 392:95. 10.1016/S0140-6736(18)31562-9 30017135

[28] Sinsky C Colligan L Li L Prgomet M Reynolds S Goeders L Allocation of physician time in ambulatory practice: a time and motion study in 4 specialties. Ann Intern Med. (2016) 165:753–60. 10.7326/M16-0961 27595430

[29] Oikonomidi T Ravaud P James A Cosson E Montori V Tran VT. An international, mixed-methods study of the perceived intrusiveness of remote digital diabetes monitoring. Mayo Clin Proc. (2021) 96:1236–47. 10.1016/j.mayocp.2020.07.040 33487438

[30] Kremer L Lipprandt M Röhrig R Breil B. Examining the mental workload associated with digital health technologies in health care: protocol for a systematic review focusing on assessment methods. JMIR Res Protoc. (2021) 10:e29126. 10.2196/29126 34342590 PMC8371485

[31] Hong YA Cho J. Has the digital health divide widened? Trends of health-related internet use among older adults from 2003 to 2011. J Gerontol. (2017) 72:856–63. 10.1093/geronb/gbw100 27558403

[32] Tomasella F Morgan HM. “Sometimes I don’t have a pulse…and i’m still alive!” interviews with healthcare professionals to explore their experiences of and views on population-based digital health technologies. Digit Health. (2021) 7:20552076211018370. 10.1177/20552076211018366 PMC8145583 34104464

[33] Choi NG DiNitto DM Marti CN Choi BY. Telehealth use among older adults during COVID-19: associations with sociodemographic and health characteristics, technology device ownership, and technology learning. J Appl Gerontol. (2022) 41:600–9. 10.1177/07334648211047347 34608821 PMC8847316

[34] Michaud TL Pereira E Porter G Golden C Hill J Kim J Scoping review of costs of implementation strategies in community, public health and healthcare settings. BMJ Open. (2022) 12:e060785. 10.1136/bmjopen-2022-060785 35768106 PMC9240875

[35] Burnes B. Emergent change and planned change—competitors or allies? The case of XYZ construction. Int J Oper Prod Manage. (2004) 24:886–902. 10.1108/01443570410552108

[36] Ferlie EB Shortell SM. Improving the quality of health care in the United Kingdom and the United States: a framework for change. Milbank Q. (2001) 79:281–315. 10.1111/1468-0009.00206 11439467 PMC2751188

[37] Cranley LA Cummings GG Profetto-McGrath J Toth F Estabrooks CA. Facilitation roles and characteristics associated with research use by healthcare professionals: a scoping review. BMJ Open. (2017) 7:e014384. 10.1136/bmjopen-2016-014384 28801388 PMC5724142

[38] Urquhart R Porter GA Grunfeld E Sargeant J. Exploring the interpersonal-, organization-, and system-level factors that influence the implementation and use of an innovation-synoptic reporting-in cancer care. Implement Sci. (2012) 7:12. 10.1186/1748-5908-7-12 22380718 PMC3307439

[39] Cummings GG Estabrooks CA Midodzi WK Wallin L Hayduk L. Influence of organizational characteristics and context on research utilization. Nurs Res. (2007) 56:S24–39. 10.1097/01.NNR.0000280629.63654.95 17625471

[40] Weiner BJ Belden CM Bergmire DM Johnston M. The meaning and measurement of implementation climate. Implement Sci. (2011) 6:78. 10.1186/1748-5908-6-78 21781328 PMC3224582

[41] Damschroder LJ Reardon CM Widerquist MAO Lowery J. The updated consolidated framework for implementation research based on user feedback. Implement Sci. (2022) 17:75. 10.1186/s13012-022-01245-0 36309746 PMC9617234

[42] The Consolidated Framework for Implementation Research. Available at: https://cfirguide.org/ (Accessed August 12, 2022).

[43] Greenhalgh T Robert G Bate S Kyriakidou O Macfarlane F. How to spread good ideas: A systematic review of the literature on diffusion, spread and sustainability of innovations in health service delivery and organisation. Available at: https://www.cs.kent.ac.uk/people/staff/saf/share/great-missenden/reference-papers/Overviews/NHS-lit-review.pdf (Accessed August 12, 2022) (2004). 10.1111/j.0887-378X.2004.00325.x PMC2690184 15595944

[44] Damschroder LJ Aron DC Keith RE Kirsh SR Alexander JA Lowery JC. Fostering implementation of health services research findings into practice: a consolidated framework for advancing implementation science. Implement Sci. (2009) 4:50. 10.1186/1748-5908-4-50 19664226 PMC2736161

[45] Nilsen P. Making sense of implementation theories, models and frameworks. Implement Sci. (2015) 10:53. 10.1186/s13012-015-0242-0 25895742 PMC4406164

[46] Wood K Giannopoulos V Louie E Baillie A Uribe G Lee KS The role of clinical champions in facilitating the use of evidence-based practice in drug and alcohol and mental health settings: a systematic review. Implement Res Pract. (2020) 1:2633489520959072. 10.1177/2633489520959072 37089122 PMC9924254

[47] Malterud K Siersma VD Guassora AD. Sample size in qualitative interview studies: guided by information power. Qual Health Res. (2016) 26:1753–60. 10.1177/1049732315617444 26613970

[48] Palinkas LA Horwitz SM Green CA Wisdom JP Duan N Hoagwood K. Purposeful sampling for qualitative data collection and analysis in mixed method implementation research. Adm Policy Ment Health. (2015) 42:533–44. 10.1007/s10488-013-0528-y 24193818 PMC4012002

[49] Atkins L Francis J Islam R O'Connor D Patey A Ivers N A guide to using the theoretical domains framework of behaviour change to investigate implementation problems. Implement Sci. (2017) 12(77):1–18. 10.1186/s13012-017-0605-9 28637486 PMC5480145

[50] QSR International Pty Ltd. NVivo (released in March 2020). Available at: https://www.qsrinternational.com/nvivo-qualitative-data-analysis-software/home (Accessed November 8, 2021).

[51] Braun V Clarke V. Using thematic analysis in psychology. Qual Res Psychol. (2006) 3:77–101. 10.1191/1478088706qp063oa

[52] Braun V Clarke V. Thematic analysis: A practical guide. London: Sage (2022).

[53] Tong A Sainsbury P Craig J. Consolidated criteria for reporting qualitative research (COREQ): a 32-item checklist for interviews and focus groups. Int J Qual Health Care. (2007) 19:349–57. 10.1093/intqhc/mzm042 17872937

[54] Damschroder LJ Lowery JC. Evaluation of a large-scale weight management program using the consolidated framework for implementation research (CFIR). Implement Sci. (2013) 8:51. 10.1186/1748-5908-8-51 23663819 PMC3656778

[55] Phichitchaisopa N Naenna T. Factors affecting the adoption of healthcare information technology. EXCLI J. (2013) 12:413–36. 26417235 PMC4566918

[56] Gagnon MP Ngangue P Payne-Gagnon J Desmartis M. m-Health adoption by healthcare professionals: a systematic review. J Am Med Inform Assoc. (2016) 23:212–20. 10.1093/jamia/ocv052 26078410 PMC7814918

[57] Qudah B Luetsch K. The influence of mobile health applications on patient—healthcare provider relationships: a systematic, narrative review. Patient Educ Couns. (2019) 102:1080–9. 10.1016/j.pec.2019.01.021 30745178

[58] ElKefi S Asan O. How technology impacts communication between cancer patients and their health care providers: a systematic literature review. Int J Med Inf. (2021) 149:104430. 10.1016/j.ijmedinf.2021.104430 PMC8131252 33684711

[59] Areia C King E Ede J Young L Tarassenko L Watkinson P Experiences of current vital signs monitoring practices and views of wearable monitoring: a qualitative study in patients and nurses. J Adv Nurs. (2022) 78:810–22. 10.1111/jan.15055 34655093 PMC9293408

[60] da Luz PL. Telemedicine and the doctor/patient relationship. Arq Bras Cardiol. (2019) 113:100–2. 10.5935/abc.20190117 31411296 PMC6684176

[61] Lee NT Karsten J Roberts J. Removing regulatory barriers to telehealth before and after COVID-19. 24.

[62] Greenhalgh T Robert G Macfarlane F Bate P Kyriakidou O. Diffusion of innovations in service organizations: systematic review and recommendations. Milbank Q. (2004) 82:581–629. 10.1111/j.0887-378X.2004.00325.x 15595944 PMC2690184

[63] Miech EJ Rattray NA Flanagan ME Damschroder L Schmid AA Damush TM. Inside help: an integrative review of champions in healthcare-related implementation. SAGE Open Med. (2018) 6:2050312118773261. 10.1177/2050312118773261 29796266 PMC5960847

[64] Edmondson AC Bohmer RM Pisano GP. Disrupted routines: team learning and new technology implementation in hospitals. Adm Sci Q. (2001) 46:685–716. 10.2307/3094828

[65] Byers V. The challenges of leading change in health-care delivery from the front-line. J Nurs Manag. (2017) 25:449–56. 10.1111/jonm.12342 26648566

[66] Howell JM Shea CM Higgins CA. Champions of product innovations: defining, developing, and validating a measure of champion behavior. J Bus Venturing. (2005) 20:641–61. 10.1016/j.jbusvent.2004.06.001

[67] Flanagan ME Plue L Miller KK Schmid AA Myers L Graham G A qualitative study of clinical champions in context: clinical champions across three levels of acute care. SAGE Open Med. (2018) 6:2050312118792426. 10.1177/2050312118792426 30083320 PMC6075611

[68] Zakerabasali S Ayyoubzadeh SM Baniasadi T Yazdani A Abhari S. Mobile health technology and healthcare providers: systemic barriers to adoption. Healthc Inform Res. (2021) 27:267–78. 10.4258/hir.2021.27.4.267 34788907 PMC8654335

[69] Broyles LM Rodriguez KL Price PA Bayliss NK Sevick MA. Overcoming barriers to the recruitment of nurses as participants in health care research. Qual Health Res. (2011) 21:1705–18. 10.1177/1049732311417727 21844286

[70] Browne S Dooley S Geraghty A Dominguez Castro P Reynolds C Perrotta C Reflections on recruiting healthcare professionals as research participants: learning from the ONSPres study. HRB Open Res. (2022) 5:47. 10.12688/hrbopenres.13499.1 36091186 PMC9428496

[71] Mehta A Mathews BK. Webside manner: maskless communication. Diagnosis (Berl). (2021) :34–9. 10.1515/dx-2020-0159 33901391

